# Nutritional Assessment of the Infant Population of the Chirikyacu Indigenous Community of Peru

**DOI:** 10.3390/nu14245217

**Published:** 2022-12-07

**Authors:** María Teresa Murillo-Llorente, Paula Montagud-Hidalgo, Javier Pérez-Murillo, María Ester Legidos-García, Alma Palau-Ferré, Marcelino Pérez-Bermejo

**Affiliations:** 1 SONEV Research Group, School of Medicine and Health Sciences, Catholic University of Valencia, C/Quevedo No 2, 46001 Valencia, Spain; 2 Department of Nursing, School of Medicine and Health Sciences, Catholic University of Valencia, C/Espartero No 7, 46007 Valencia, Spain

**Keywords:** anthropometry, malnutrition, health status, nutritional status, child nutrition, Peru, indigenous communities

## Abstract

Malnutrition is one of the main public health problems affecting early childhood development, compromising the health and quality of life of thousands of Peruvian children. The main contribution of this work is the analysis of the nutritional status of the infant population of the Chirikyacu Indigenous Community of Peru in order to evaluate current and future food policies. It is a cross-sectional study with a sample of 19 children between 6 months and 6 years of age. Sociodemographic, anthropometric, clinical variables and dietary habits were analyzed from 7 January to 4 February 2018. The mean age of the children was 29.74 months (SD = 23.91). We found statistically significant differences between the BMI values of boys and girls (*p* < 0.05; Mann–Whitney test). According to the z-scores, 35.29% suffer from malnutrition, although in no case is it severe. Hair, nails and skin were in good condition in general, except for some cases showing signs of nutritional deficiency. Dietary patterns are based on legumes, vegetables, dairy products, ice cream, cookies and sweets, and are considered insufficient to cover basic needs; water intake was also insufficient. Breastfeeding lasted an average of 14 months (SD = 2.9). We found a prevalence of malnutrition higher than that established by the WHO in Latin America among the children of Chirikyacu. The dietary pattern is insufficient to cover basic needs, so it is necessary to continue with nutritional educational interventions to improve it.

## 1. Introduction

Nutritional status determines health, cognitive and psychosocial development in early childhood. Regardless of the cultural and socioeconomic context, human beings require adequate nutritional conditions for optimal development [1]. Observing nutritional deviations allows therapeutic and preventive decisions to be made so that children grow properly [2] avoiding the onset of diseases [3], since an adequate nutrient intake produces a growth hormone mediating effect [4].

Child nutritional assessment is based on family history, clinical examination and anthropometry. In addition it is helpful to be aware of the diet, eating behavior, type of physical exercise performed frequently and pathologies that affect nutrition [2]. There are many pathologies associated with inadequate nutrition, such as diarrhea, infections and excess weight [5], which are responsible for diseases and deaths.

In 2009, the Peruvian Ministry of Health, the Peruvian Ministry of Women and Social Development, the United Nations Children’s Fund in Peru and the United Nations World Food Program in Peru launched the Multimicronutrient Implementation Plan (MMN) 2009–2011 [6], in order to prevent and control nutritional problems in children aged 6–35 months through supplementation with MMN powder known locally as “Chispitas”, reducing the incidence of anemia by 51.7% [7]. It is a supplementation to prevent and treat mineral and vitamin deficiencies, made up of vitamin A, folic acid, acetate, vitamin C, zinc, ascorbic acid, zinc gluconate, iron, ferrous fumarate (C4H2FeO4) and maltodextrin, which is mixed with the child’s semi-solid food [8,9].

According to the 2020 Demographic and Family Health Survey (ENDES) [10], in 2015, 14.4% of the Peruvian child population was found to be undersized for their age, with chronic malnutrition affecting children in rural areas to a greater extent (27.7%), 18.5 percentage points more than in urban areas (9.2%). In 2020, the World Health Organization (WHO) reported more encouraging malnutrition figures, 12.1% in children under 5 years of age. Comparing 2009 with 2020, malnutrition has decreased, thus reducing the difference between the urban and rural population, although it is still very characteristic.

Iron-deficiency anemia is most common in rural areas of Peru [11]. According to the 2020 ENDES survey [10], it affects 40.0% of children aged 6–35 months. It is important to perform an assessment of nutritional status to know the alterations that may affect the health of children and to treat them prematurely, as well as to perform a clinical-nutritional study with a physical examination, anthropometry and body composition [12].

Acute diarrheal disease (AD) is responsible for child deaths, especially in impoverished environments. In 2020, the prevalence in children up to 5 years of age was 8.2%, with more infants (6–23 months) suffering from it. The results of the 2020 ENDES survey [10] revealed a prevalence of childhood diarrhea of 8.2%, showing a decrease from 2015 (12.0%).

The Chirikyacu community is a native Lamista community located in the high jungle of the Peruvian Amazon who live self-sufficiently. The Amazon jungle is partly lush and partly deforested. The houses do not have the minimum sanitary conditions; they have latrines because they do not have sanitary sewers. The roofs of the cabins are made of palm. They have had electricity since 2013, so they can listen to music and watch satellite television.

The community contains men under 45 years of age. From this age onwards they are considered elderly, of which there are about 10 people. These are dedicated to the care of their “chacras”, rural properties of reduced extension designated for growing crops and as places of rest. The men work in the community under the orders of the “Apu”, who is the person elected every 2 years by all members and who represents the highest authority.

The child population is scarce because some women take a concoction with a contraceptive effect composed of snake venom known as “viborachado” [13] and most of them are given a contraceptive injection every 3 months. The number of children per family is usually two or three. The children are cheerful, alert and curious. They play soccer every day, run around the hillside, climb trees and go to the farms with their mothers to help them with the work in the vegetable garden, most of the time barefoot, and although they bathe every day, they are apparently dirty, with muddy remains due to the conditions of the terrain. The physical activity of the children during the day is high. Compulsory education ends at the age of 11, and there is a kindergarten and a small school.

The women work more than the men in the community; they pick coffee on the farm (where the scarce income comes from), make clay pots, weave “cinchas” (a type of belt), cook, take care of the children and take care of the firewood. They cook on improvised stoves or “candelas” inside the house. The older women work with clay and weavings, teaching the trade to future generations. The women must obey their husbands’ orders.

The community has a hospital 45 km away by car, in the town of Lamas, and locally there is a health post with minimal resources, staffed by a health technician (open only a few days a month). The road is unpaved and practically impassable during the rainy season.

Díaz and collaborators [14] state that it is necessary to carry out nutritional assessments of indigenous populations because their living conditions and health resources are scarce and, if necessary, to implement strategies to improve their level of health. In Chirikyacu they have improved their habits thanks to communal programs [15].

In view of the above, the aim of the present study was to analyze the nutritional status of the children in the native community of Chirikyacu, in the Amazon jungle. A correct assessment will allow the identification of alterations and will make possible a therapeutic approach. The research questions we asked ourselves were: What is the nutritional status of the children living in the indigenous community? What are their anthropometric characteristics? Is their growth adequate? Lasty, what is their dietary pattern?

## 2. Materials and Methods

This was a cross-sectional study. Data were collected between 7 January and 4 February 2018. Children whose age was between 6 months and 6 years, residing in the community of Chirikyacu, Peru at the time of data collection were included. Children who were ill at the time of data collection or had chronic pathologies or disabilities were excluded. The study was conducted using the direct survey method known as PAPI (Paper and Pen Personal Interview). No sampling was performed. The final sample consisted of all the children living in Chirikyacu at the time of data collection, a total of 19 children.

### 2.1. Ethical Approval and Consent to Participate

The project obtained a positive opinion from the UCV Research Ethics Committee (approval code UCV/2017-2018/27), and written informed consent was obtained from all the participants. This study complies with the principles set forth in the Declaration of Helsinki [16].

### 2.2. Variables

Sociodemographic, anthropometric, clinical and dietary habit variables were analyzed. To collect dietary data, a validated instrument was used [17]. This was a questionnaire on dietary knowledge and eating habits with good inter-observer agreement (0.5–1.0) and high internal consistency (0.75) and test–retest correlation (0.6–1.0).

For anthropometry, each child’s weight and height were measured at the same time in the morning, on an empty stomach, while wearing underwear using precision balanced scales and measuring rods appropriate to the age of the child,. Using a flexible tape measure, brachial, waist and hip perimeters were measured to determine central obesity [18]. With the data obtained, BMI and waist/height and waist/hip ratios were calculated. Finally, all the children underwent an examination of the skin, nails, mucous membranes, hair and teeth.

### 2.3. Statistical Analysis

The data are presented using statistics of central tendency and dispersion: mean and standard deviation (SD). The data corresponding to qualitative variables are expressed as the absolute value of cases and in percentage (%). The normality of the data was studied with the Kolmogorov–Smirnov goodness-of-fit test. The differences between the quantitative variables were analyzed using the Mann–Whitney U test and the Kruskal–Wallis H test, and their correlation with Pearson’s coefficient. The association between the qualitative variables was studied using the Chi2 test. The collected data were analyzed using SPSS (Statistical Package for Social Sciences) version 23 (SPSS Inc., Chicago, IL, USA). The z-scores for weight, height and BMI were obtained using lmsGrowth© software, a Microsoft Excel add-in developed by Tim Cole, University College London, freely downloadable from https://www.healthforallchildren.com using reference WHO2007 (accessed on 10 November 2022). A significance value < 0.05 was accepted for all tests.

## 3. Results

A total of 19 children were included in the study, of whom 8 were male (42.1%) and 11 female (57.9%). The mean age was 29.74 months (SD = 23.91) and half of the children were younger than 18 months. The maximum value was 72 months and the minimum was 6 months. No statistically significant differences were found between the age of males and females (*p* > 0.05; Mann–Whitney test). Table 1 shows the anthropometric characteristics of the entire infant population of the Chirikyacu community differentiated by sex.

We found statistically significant differences between the BMI values of boys and girls in the study sample (*p* < 0.05; Student’s t-test). Boys had a BMI 1.32 units higher than girls (95%CI 0.4–2.25). All parameters were within the normal range for growth and no child with a BMI above or below the normal range was observed. Weight, height and BMI z-scores were calculated for all the children in the community. Table 2 shows a summary of the results obtained.

We found 15 children of normal weight for their age, 1 at risk of global malnutrition, 1 with moderate malnutrition and 1 with severe malnutrition. Regarding height, 13 children were of normal height, 3 were of decreased height typical in children with chronic malnutrition, 2 were of a height indicating moderate chronic malnutrition and 1 was of a height indicating severe chronic malnutrition, namely a 4-month-old boy born at term (37 weeks of gestation).

Analyzing the BMI, 1 child (case 15) was at risk of obesity with a waist/hip ratio of 0.96, a value compatible with android syndrome, and a waist/height ratio of 0.59, indicating a risk of suffering cardiovascular or metabolic disease [19]; 14 children were within normal values, 3 were at risk of acute malnutrition, 1 was at risk of moderate-acute malnutrition and none of them suffered from severe-acute malnutrition.

### 3.1. Clinical Nutritional Aspects

Table 3 shows the clinical aspects related to nutrition observed in the analyzed child population.

In 26.31% of the population (*n* = 5), the skin was dry, rough and dehydrated, with bite marks and skin lesions. Exposure to the sun is high, they do not use moisturizing creams or oils and the soap used for showering is the same as the one used for washing clothes. Most of the children’s hair was in good condition; the rest were characterized as having sparse or fuzzy hair. The condition of the nails was generally good. It was observed that they wash their hands before meals as they have participated in interventions to improve their lifestyle habits. Regarding the mucous membranes, we observed that all children had normal or hydrated mucous membranes. Finally, regarding the dentition, some of them presented dark stains which could be tartar due to lack of hygiene or the presence of chromogenic bacteria. Caries or yellow stains were also observed (closely related to tetracycline treatments). In general, the children brushed their teeth very little because, although they all had toothbrushes at home, they did not have toothpaste. On the other hand, they drink coffee from the age of 1, and their teeth may darken due to this circumstance.

### 3.2. Eating Patterns

Regarding food cooking, the women usually cook the food they grow themselves on a daily basis. The way they prepare food is “sancochada” (a word that indicates al dente cooking) in “candelas” (wood fires), roasted (mainly pork), or fried. Few stews are prepared, and salt consumption is high. Although the government has offered them the possibility of building an improved kitchen with a chimney to avoid the concentration of smoke particles in the house, which could affect their health, they continue to cook with firewood. Daily meals usually number two or three, although when they have an appetite they eat nuts, fruit or bread. They do not usually keep a strict schedule, although they have breakfast at around 7 a.m., lunch at 12 p.m. and dinner at 7 p.m. The enormous educational deficiencies of the mothers related to food and diet, which is very repetitive, have been observed.

Sugar cane is highly consumed, as well as honey (produced by bees). Hunting has decreased in the area, so they have less high-protein food. Families usually have laying hens that provide them with eggs on a daily basis. The type of oil consumed is palm oil. Table 4 shows the dietary patterns. It is important to point out that, although the survey was aimed at the child population, since the children were so young, the mothers responded directly about the dietary pattern of their children.

Among the vegetables they eat are cucumber, broccoli and cauliflower. Many mothers dress and mix vegetables at least two times a week and usually do so with palm oil and lemon. They do not usually make sandwiches. The bread sold in the community is a rounded bread roll at a cost of 0.10 soles (EUR 0.20) and not everyone can afford it. Those who said they prepare it usually fill it with canned tuna.

The fruits available are pineapple, banana, papaya, tomato, orange and tropical fruits. Fruit is one of the main foods they grow in Chirikyacu, especially bananas (ripe, manzanita and guineo). All the families have “chacras” (an American term to identify the land allocated by the community to grow different foods for subsistence and sale), so they usually have seasonal fruits, and there are even wild trees with fruits such as mango from which the children pick the fruit directly and eat it. Part of their income is due to the sale of what they harvest in the town of Lamas, 45 min away by car. Regarding the answers about fruit consumption, we think that the answers are not true because they eat bananas every day at every meal. Chirikyacu grows a lot of cocoa, a product that is consumed in abundance mixed with coffee. Another widely consumed food is raw peanuts, which provide them with many benefits.

In the community most of the children eat at home, as many families cannot afford to pay for school meals. Only a minority eat at school.

Water is obtained from the mountains, arriving through pipes, and in times of rain it is cloudy, even with small stones, therefore, it is not drinkable, and it is boiled or drawn from a well for consumption. This is one of the reasons why they suffer from diarrhea, as the water contains parasites that cause amoebiasis. The daily intake of water is low, and they usually boil it with fruit or citrus fruits such as lemon, or coffee, adding a large amount of sugar.

Most children said they eat between one and three plates of vegetables or salad a day. This answer is questionable after looking directly at what their diet is based on. Chirikyacu produces vegetables and the nearby community, Chunchiwi, grows lettuce which they sell, give away or exchange with some families in Chirikyacu.

In Chirikyacu they started in 2018 to make bread thanks to a state project. The sale of bread continues to increase. Before 2018, it was not a food incorporated into the diet. A lot of the population does not consume bread since they do not have money to buy it. Children receive bread with coffee. The bread has a high sugar and butter content, in addition to corn flour. Families eat cereals such as corn, which provides minerals (copper, iron, magnesium, phosphorus and zinc) and vitamins (A, B, C, E). They usually prepare it in a drink known as chicha (fermented corn) or also in the mazamorra that they prepare at parties.

Very few children eat sweets. In the community there are two small stores inside two houses where they sell some of these products, and it is the children of the wealthiest families who usually buy them. They are not big consumers of sweets, amongst other things because they do not have them (only sporadically and on birthdays).

In the community they do not raise animals that offer them the possibility of having milk. An added difficulty is that they do not have refrigerators, except for one family. Most of the population consumes one portion of dairy per day, which is usually buttery cheese. The fresh fish they obtain is transported in trucks to the community, although they usually eat salted fish or canned tuna.

In the community they usually drink Inca Kola, a very popular drink among the Peruvian population, rich in sugar (21 g) and sodium (18 micrograms). The children drink it whenever they have money.

Finally, many children consume ice cream once a week, as an alternative to snacks and lunches, also as snacks between meals. What they usually consume are the so-called “chupetes” which are ice creams made with crushed fruit.

### 3.3. Breastfeeding

The mean duration of breastfeeding was 13.88 months (SD = 2.9) and half of the children were breastfed for more than 14 months. The maximum value was 18 months and the minimum was 7 months. No statistically significant differences were found with respect to the mother’s profession or the number of siblings (*p* > 0.05; Kruskal–Wallis test).

We found a positive correlation between the duration of breastfeeding and the current weight and height of the children (*p* < 0.001; Pearson test). Children who breastfed for longer had higher weight and height values. The regression equations explain 48.5% (weight) and 45.8% (height) of the variability, which is considered acceptable for a small sample.

No association was found between the duration of breastfeeding and the condition of the skin, hair, nails, mucous membranes or dentition (*p* > 0.05; Chi-square test).

## 4. Discussion

Peru is a country with wide social and regional disparities [20]. In this study we have analyzed the nutritional status of children up to 6 years of age in an Indigenous community of that country, the native community of Chirikyacu, in the Amazon jungle, since a correct assessment will allow the identification of alterations and will enable a therapeutic approach. Any factor that alters the nutritional status in the first years of life will have negative repercussions on health and, therefore, periodic monitoring is the best strategy for detecting nutritional alterations and evaluating children’s development [21].

In order to identify waist-to-hip ratios (WHR), the WHO recommends the following values: a normal WHR for women ranges from 0.71 to 0.84; normal values for men range from 0.78 to 0.94 [22]. Waist and height are independent measures of the associated risk, but hip circumference does not show discriminatory power [23]. A study conducted in Bogotá [24] considered that subjects whose waist/hip ratio was above the 90th percentile of the standard normal distribution were at high cardiovascular risk (boys ranging from 0.87 to 0.93 and girls from 0.85 to 0.89). In general, the waist/hip ratio values were lower than values in Europe, Asia and Africa and similar to those of some Latin American references; however, in our study we observed that the waist/hip ratio values tended to be higher than those described, making it necessary to adequately follow up these children to prevent cardiovascular risk with health promotion activities. In contrast to what was described in the 2020 ENDES survey [10], the rest of the anthropometric values were found to be within the normal range, so we cannot affirm that chronic malnutrition exists in this community.

In our study the degree of nutrition of the community was evaluated by calculating z-scores with the aim of comparing the results with the WHO decreasing patterns [25]. In terms of z-height it was observed that most of the children were between −1SD and + 1SD, so we understand that the children of the community are of an appropriate height for their age according to the WHO recommendations, although we must take into account that we found children with scores compatible with global, moderate, and in one case, severe malnutrition.

With respect to the z-weight and BMI score, we observed many children with a score between the normal limits. Importantly, we found three children at risk of acute malnutrition and one at risk of moderate-acute malnutrition. Malnutrition (35.29%) in the children of this community was higher than the results established by the WHO [25] and the Demographic and Family Health Survey [10]. These data are similar to those of the Technical Report on Nutritional Status in Peru published by the Peruvian Ministry of Health [26], which states that malnutrition is usually three times higher in rural areas than in urban areas.

Malnourished children who were identified should be monitored by local health workers and micronutrient intake should be recommended. Although some studies, such as Penny’s [27], conclude that these are not sufficient to stop malnutrition, others consider them effective from 2 months of intake [7,8,9]. However, in the Chirikyacu community, some mothers throw them away because of negative beliefs.

The clinical-nutritional aspects of our results provide evidence of malnutrition [28], such as thin skin and a weak pulse. However, despite finding some cases of malnutrition, we observed that most of the children were characterized by a good skin condition, although their hair was thinner and lighter than is typical. In children with malnutrition, we found a thin and dry skin, which could be due to lack of vitamin A, niacin and vitamin B6 [29]. Vomiting, diarrhea and infectious diseases are clinical signs of malnutrition [30]. In our study only one child was found with a pathological history of diarrhea and the other children did not suffer from any previous pathology.

Another clinical aspect of malnutrition to highlight is hair which was fine, sparse and easily shed, as well as dry, dull and hypopigmented [29]. We observed children with hair in good condition and adequate for the population of Peru, although a small percentage of the population had thin hair. These children should be the subject of an in-depth study to find the origin of the condition of their hair, since children with malnutrition have a deficiency of vitamin A, zinc and biotin [30].

Regarding dietary patterns, we observed that among the child population of Chirikyacu the diet is extensive and is based on the consumption of products grown on the farms. The children eat mainly rice, plantains and legumes. It is an area where agriculture is the basis of the economy, since the “Apu” (person chosen by the community to maintain internal order) offers each family member a farm to cultivate and produce basic food for their diet and economic supply, thus turning the community’s diet into a sociocultural phenomenon [31]. We have observed that the food pattern of the population is repetitive: rice, beans, bananas, coffee, corn and tropical fruits. A typical dish of the men who go into the jungle is “juanes” (meat with rice and spices cooked in banana leaf), or the “inchicapi broth” made of Amazonian chicken, with peanuts and coriander.

Most schoolchildren consume two or more foods a week rich in sugar and/or fats, results similar to the study by Aparco and collaborators [32] where children attending school canteens consumed cookies, chocolates, salty snacks, commercial juice and soft drinks between two and three times a week. In the Chirikyacu community sugar-sweetened beverages are consumed at all meals. The basic complements to their meals are rice, legumes, banana and a bread roll, a source of complex carbohydrates that provide energy [31].

In the previously mentioned study [32], it was observed that 68% of school children consumed fried foods and 52% consumed cookies two or more times per week. We observed that most children in the Chirikyacu community (57.9%) do not consume this type of food. Regarding protein consumption, the main food was fish. Fish, thanks to its contribution of omega-3 fatty acids, contributes to a reduction in cardiovascular risk, which is why it is advisable to include it in the family diet twice a week or more [31]. A food that should also be highlighted for its high biological value is milk, consumed once a day, although we believe that these responses are biased, since, during the stay of the research team, they did not consume fish or milk as indicated. Vegetables and salads are not lacking in the diet of this community, complying with the WHO recommendations [30].

It is important to prolong breastfeeding until 24 months for its benefits [33,34]. Breast milk is the optimal food for infants during the first year of life [35]; however, some studies show that a very high percentage of infants discontinue breastfeeding before 6 months because the baby does not gain weight [36,37]. However, we observed that the babies in Chirikyacu were breastfed for a mean duration of 13 months (SD = 2.9), a very adequate time according to the WHO, although it would be advisable to extend it further, since dairy products are difficult to obtain in the community and, in addition, the results are in line with other studies [38,39] that conclude that the prolonged duration of breastfeeding benefits growth, physical-affective development and intelligence, and decreases the prevalence of pathologies in both the child and the mother.

The main limitation of our study is the low participation of the infant population because they were on vacation outside the community. Furthermore, the responses may be biased, since almost 30% of the population is illiterate [15] and they have difficulties in understanding the Spanish language because they are Quechua-speaking people. On the other hand, the strength of our work lies in the direct observation by the research team over a long period of time of the food habits of the community.

## 5. Conclusions

We found a prevalence of malnutrition higher than that established by the WHO among children of the Indigenous community of Chirikyacu. Although the anthropometric characteristics in a high percentage of the population studied are within the normal ranges established by the WHO, cases of global, moderate and severe malnutrition were found. The children in the community did not present relevant pathological antecedents and the clinical nutritional findings showed cases of nutritional deficiency. The dietary pattern of the community is insufficient to cover the basic needs of the children, based on beans, rice, eggs, bananas, cocoa, coffee and ultra-processed products. It is necessary to continue with nutritional educational interventions to improve their dietary patterns.

## Figures and Tables

**Table 1:** Anthropometric characteristics of the infant population of the Chirikyacu community.

	Gender	*n*	Mean	SD *	*p*-Value **
Weight (g)	Male	8	12,338	4615	0.771
	Female	11	11,594	5891	
Height (cm)	Male	8	85.8	17.1	0.911
	Female	11	84.7	22.4	
BMI	Male	8	16.36	1.00	0.007
	Female	11	15.04	0.89	
Head circumference (cm)	Male	8	46.2	2.1	0.252
	Female	11	44.0	4.9	
Arm circumference (cm)	Male	8	14.8	1.6	0.679
	Female	11	14.4	2.0	
Waist circumference (cm)	Male	8	47.4	5.8	0.641
	Female	11	45.7	8.5	
Hip circumference (cm)	Male	8	49.0	7.0	0.542
	Female	11	47.0	10.0	
Biceps skinfold (mm)	Male	8	9	4	0.217
	Female	11	11	4	
Triceps skinfold (mm)	Male	8	9	4	0.314
	Female	11	11	3	
Subscapular skinfold (mm)	Male	8	7	2	0.990
	Female	11	7	2	
Suprailiac skinfold (mm)	Male	8	4	2	0.261
	Female	11	5	1	

* SD: Standard Deviation; ** Mann–Whitney test.

**Table 2:** Summary of weight, height and BMI z-scores of the children in the study.

	z Height	z Weight	z BMI
>3 SD	0	0	0
>2 SD	0	0	0
>1 SD	3	0	1
Between −1 and +1	10	15	14
<−1 SD	3	1	3
<−2 SD	2	1	1
<−3 SD	1	2	0
Total	19	19	19

**Table 3:** Clinical-nutritional aspects of the children in the study.

		N (%)
Pathological history	No pathology	16 (84.21)
	Fever due to vaccination	1 (5.26)
	Diarrhea	1 (5.26)
	Bronchitis	1 (5.26)
Skin condition	Good	13 (68.42)
	Dry	4 (21.05)
	Dehydrated	1 (5.26)
	Impetigo	1 (5.26)
Hair condition	Good	16 (84.21)
	Sparse	3 (15.79)
Condition of nails	Normal	17 (89.47)
	Thin	1 (5.26)
	Soft	1 (5.26)
Condition of mucous membranes	Moisturized	19 (100)
Condition of dentition	None	3 (15.79)
	Normal	11 (57.89)
	Caries/stains	5 (26.32)

**Table 4:** Dietary patterns in the child population in the study.

		N (%)
Mix and dress vegetables	Never	1 (5.26)
	1–2 times per week	12 (63.16)
	3 or more times per week	4 (21.05)
	NA	2 (10.53)
Make a sandwich	Never	8 (42.11)
	1–2 times per week	6 (31.58)
	3 or more times per week	2 (10.53)
	NA	3 (15.79)
Peeling and chopping fruit	Never	2 (10.53)
	1–2 times per week	6 (31.58)
	3 or more times per week	9 (47.37)
	NA	2 (10.53)
Daily fruit consumption	1 piece	5 (26.32)
	2 pieces	5 (26.32)
	3 pieces	2 (10.53)
	4 or more pieces	4 (21.05)
	NA	3 (15.79)
Preparing a plate of food alone	Yes	10 (52.63)
	No	5 (26.32)
	NA	4 (21.05)
Where do you eat breakfast?	Home	14 (73.68)
	NA	5 (26.32)
Where do you eat lunch?	Home	11 (57.89)
	College	3 (15.79)
	NA	5 (26.32)
Glasses of water you drink per day	1 glass	6 (31.58)
	2 glasses	6 (31.58)
	3 glasses	1 (5.26)
	4 or more glasses	3 (15.79)
	NA	3 (15.79)
Daily vegetable/salad dishes	1 plate	13 (68.42)
	3 plates	2 (10.53)
	Does not eat	2 (10.53)
	NA	2 (10.53)
Daily Consumption of marraqueta or hallulla breads	Does not eat	5 (26.32)
	Half a loaf of bread	2 (10.53)
	1	6 (31.58)
	4 or more	3 (15.79)
	1 per week	1 (5.26)
	NA	2 (10.53)
Consumption of french fries, pizzas, sopaipillas per week	Does not eat	11 (57.89)
	1	3 (15.79)
	2	1 (5.26)
	More than 4	2 (10.53)
	NA	2 (10.53)
Consumption of dairy products per day	Does not eat	4 (21.05)
	1	10 (52.63)
	2	1 (5.26)
	More than 4	2 (10.53)
	NA	2 (10.53)
Consumption of fish per week	Does not eat	3 (15.79)
	1	6 (31.58)
	2	6 (31.58)
	3	1 (5.26)
	More than 4	1 (5.26)
	NA	2 (10.53)
Daily consumption of juices/carbonated beverages	Does not eat	7 (36.84)
	1	4 (21.05)
	2	3 (15.79)
	3	2 (10.53)
	Sometimes 1/week	1 (5.26)
	NA	2 (10.53)
Weekly consumption of legumes	Does not eat	1 (5.26)
	1	2 (10.53)
	2	2 (10.53)
	3	1 (5.26)
	4 or more	11 (57.89)
	NA	2 (10.53)
Weekly consumption of ice cream, cookies and sweets	Does not eat	4 (21.05)
	1	8 (42.11)
	2	2 (10.53)
	3	1 (5.26)
	4 or more	2 (10.53)
	NA	2 (10.53)

NA: No Answer.

## Data Availability

Not applicable.
